# A Comprehensive Analytical Approach for Quality Control of Collagen in Food Supplements

**DOI:** 10.3390/md22100435

**Published:** 2024-09-26

**Authors:** Nika Kržišnik, Ema Kurent, Robert Roškar

**Affiliations:** Faculty of Pharmacy, University of Ljubljana, Aškerčeva Cesta 7, 1000 Ljubljana, Slovenia; nika.krzisnik@ffa.uni-lj.si (N.K.); emkurent@gmail.com (E.K.)

**Keywords:** marine collagen, anti-aging, food supplements, quality control, HPLC-UV, spectroscopic methods

## Abstract

Collagen is a popular nutricosmetic ingredient in food supplements due to its anti-aging and other positive effects on the skin. Due to its widespread use and the lack of regulation in this area, appropriate quality control is required to ensure efficacy and safety, with the development of analytical methods playing an important role. Currently, the quantitative determination of collagen is mainly based on time-consuming derivatization-based spectroscopic methods or on complex chromatographic methods with mass spectrometric detection. Therefore, in this study, two new, simple chromatographic methods have been developed. One is intended for the analysis of untreated samples and is characterized by the speed and simplicity of sample preparation. The other method quantifies collagen via the underivatized tripeptide Gly-Pro-Hyp formed by bacterial collagenase hydrolysis and is characterized by its specificity and ability to distinguish between marine and terrestrial collagen. The latter is a novelty in the field of simple methods for collagen analysis and is particularly important in terms of safety. Our comparison with established analytical methods (e.g., via hydroxyproline after complete hydrolysis) for collagen analysis undoubtedly showed the superiority of these new methods for the routine quality control of collagen supplements in terms of specificity, repeatability, sample stability, and simplification in sample preparation. The collagen content in the supplements tested was found to be adequate; however, some discrepancies were found regarding the labeling and origin of the collagen, with possible safety implications.

## 1. Introduction

Collagen and its hydrolysates are widely used in the food industry, medicine, pharmacy, cosmetics, and tissue engineering due to their unique properties [1]. Currently, collagen is a popular nutricosmetic ingredient due to its anti-aging and other beneficial effects on the skin [2,3,4]. Orally administered collagen hydrolysate improves skin physiology and appearance by increasing skin hydration, elasticity, and firmness, reducing wrinkles and hyperpigmentation, promoting wound healing, and rejuvenating the skin. These anti-aging effects are achieved through its antioxidant and anti-inflammatory action, water retention, promotion of the proliferation and growth of fibroblasts, which are important for the synthesis of the skin’s own collagen, and inhibition of elastases and metalloproteinases, which cause the breakdown of elastin and collagen, respectively [1,2,3,4,5,6].

Hydrolyzed collagen for oral supplementation can be obtained from tissues such as the bones, cartilage, tendons, and skin of various animal species. For many years, bovine and porcine collagen were the only sources of collagen. However, due to the outbreak of various diseases and religious restrictions, alternative biocompatible collagen sources with low immunogenicity, such as marine collagen, are increasingly being used [1,2,7]. In addition to safety, the use of marine collagen offers the advantage of better digestibility, a faster absorption rate, and better bioavailability [6]. Marine collagen is also easier to hydrolyze and process into low-molecular-weight collagen peptides, which have a better antioxidant effect, are more water soluble, and are absorbed faster [1,3,8]. The extraction of marine collagen is also environmentally friendly, as it can be obtained from by-products of the food industry in high yields and at low cost [1].

In contrast to medicinal products, there is a lack of regulations in the area of food supplements and cosmetics. Due to the popularity and widespread use of anti-aging products, appropriate quality control is required to ensure their efficacy and safety, as products with inappropriate contents of active ingredients, mislabeling, inappropriate daily doses, the presence of impurities, contaminants, pharmacologically active or toxic substances, and adulteration are often found on the market [9,10,11,12,13,14,15,16,17,18,19]. One such example is resveratrol, as products with a lower content are often marketed, resulting in lower efficacy [9]. On the other hand, products are also often found to exceed the legally permitted maximum levels, which poses health risks [10]. The purity of the resveratrol is also important for the anti-aging effect, as multi-ingredient products are less effective [9]. In addition, products are often mislabeled, contain misleading health claims, and provide information that misleads consumers [10]. Misleading claims are also common with quercetin products. The recommended daily doses are often very high, which can lead to clinically relevant drug interactions causing safety issues [11]. Products containing luteolin are often adulterated, as luteolin is mixed with large amounts of the less effective and much cheaper quercetin glycoside rutin. Furthermore, frequently, neither the purity nor the source of the luteolin is labeled, which is problematic from a safety point of view. In addition, the products are often poorly formulated, resulting in low absorption and, consequently, poor efficacy [12]. Food supplements containing turmeric often do not contain the labeled amounts of curcuminoids, and the composition of the curcuminoids does not meet the pharmacopeial requirements, indicating the unlabeled use of synthetic curcumin. Labeling issues are also found with these products. In addition, these products have been found to contain active ingredients that may alter the metabolism of concomitant medications, as well as heavy metals and toxic organic solvents, which has safety implications [13,14]. Food supplements containing coenzyme Q10 often do not contain the specified amount or the specified form (ubiquinol vs. ubiquinone). Both issues are associated with insufficient stabilization. The chemical form of Q10 and the dosage form are important for its bioavailability and, consequently, for its efficacy [15]. Similar issues are also observed in cosmetic anti-aging products containing coenzyme Q10, vitamins A and E, and/or the B-complex. The instability of these ingredients and insufficient stabilization often lead to a lower content of active ingredients. Labeling irregularities are also frequently observed. The addition of prohibited ingredients, which is associated with health risks, is also problematic [16,17,18,19].

A literature review revealed a lack of data on the quality of collagen-containing supplements, which is why the development of appropriate analytical methods and product quality control are important to ensure the efficacy and safety of these products. The efficacy of collagen-containing supplements depends on the collagen content and the degree of its hydrolysis, while the origin of the collagen and possible residues of toxic organic solvents used in the extraction process are important for their safety [5,6,8,20]. We have noticed that despite the relevance of this topic, no new approaches for collagen analysis have been developed recently. In general, various complementary analytical techniques, such as spectroscopic, immunochemical, and chromatographic, are used [20,21,22,23], and the analysis of collagen is based on its unique structure as different types of collagen share a common structural motif: a right-handed triple helix consisting of three left-handed alpha chains. The triple helix domains consist of a repeating sequence, Gly-X-Y, where X and Y are often proline (Pro) and 4-hydroxyproline (Hyp), respectively. Hyp is formed during post-translational modifications and is an almost unique feature of collagen, as it is very rare in other proteins [24]. The latter is the basis for the most commonly used method for the quantification of collagen as it is based on the acid or base hydrolysis of collagen to amino acids, whereby the resulting Hyp is first oxidized and then derivatized with Ehrlich’s reagent [21,25]. An alternative colorimetric assay uses an anionic red dye, Sirius-Red, which binds the basic amino acid residues in collagen [22,23]. Collagen can also be quantified fluorometrically after derivatization with o-phthalaldehyde (OPA) [22,26] or after enzymatic hydrolysis with bacterial collagenase and complexation of the resulting N-terminal glycine-containing peptides (NGPs) with 3,4-dihydroxyphenylacetic acid [22]. Amino acids resulting from the complete hydrolysis of collagen can also be derivatized with reagents to form fluorescent products and allow the quantification of collagen by reversed-phase high-performance liquid chromatography (RP-HPLC) [27,28,29], whereas amino acid analyzers with automatic post-column derivatization [30] or more complex RP-HPLC methods with mass spectrometric detectors have to be used for non-derivatized samples [23,31,32,33]. Size-exclusion chromatography (HPLC-SEC) is used to determine peptide and protein size in collagen samples [20,34,35,36].

Since the quantitative determination of collagen is currently mainly based on time-consuming and non-selective spectroscopic methods or on complex chromatographic methods based on derivatization or mass spectrometric detection, the main objective of our work was the development of contemporary, simple, and fast HPLC methods with UV detection for the evaluation of collagen content in food supplements, focusing on the ability of these methods to distinguish marine from terrestrial collagen. The developed methods were compared with established spectroscopic methods commonly used in collagen analysis. The suitability of the validated analytical approach for the quality control of commercially available collagen-containing food supplements was further evaluated.

## 2. Results

### 2.1. Method Development

**Figure 1:**
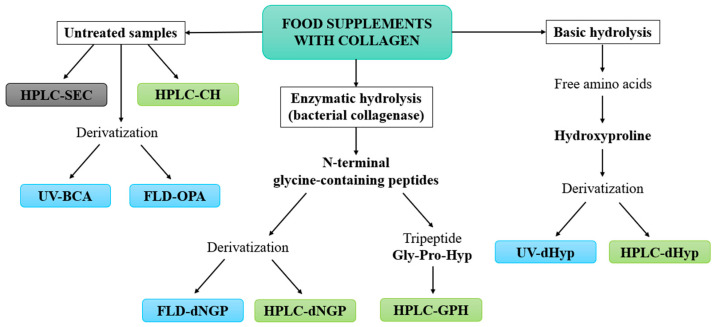
Workflow for the development of chromatographic methods (green color) for the quantification of collagen in food supplements. The methods were compared with spectroscopic reference methods (blue color), namely the FLD-OPA, UV-BCA, UV-dHyp [21], and FLD-dNGP [22]. The qualitative HPLC-SEC method (gray color) was used to evaluate the peptide size in collagen products.

The development of novel HPLC methods for the quantification of collagen in food supplements was based on three main concepts (Figure 1). The basis was the most commonly used method for the quantification of collagen via Hyp. We investigated whether the specificity of the established spectroscopic method (UV-dHyp) [21] could be improved by chromatographic separation (HPLC-dHyp). The second group of methods was based on NGPs, which are formed after the enzymatic hydrolysis of collagen with bacterial collagenase. Here, too, we investigated whether the use of chromatography (HPLC-dNGP) offers an advantage over the reference spectroscopic method (FLD-dNGP) [22]. In addition, the goal was to develop a method for the determination of collagen via the underivatized tripeptide Gly-Pro-Hyp (HPLC-GPH), which is the most abundant tripeptide formed during the enzymatic hydrolysis of collagen. The third concept was the development of methods for the analysis of untreated samples of food supplements containing collagen hydrolysates (HPLC-CH). In addition, the possibility of using simple and established spectroscopic techniques for the evaluation of protein concentration, namely the FLD-OPA and UV-BCA methods, was also investigated. The Bradford method was also tested, but proved to be unsuitable due to its low sensitivity, as collagen contains very few aromatic and basic amino acid residues. The HPLC-SEC method was only used to evaluate the size of the peptides in collagen products and is therefore a qualitative method.

The development was initiated with the HPLC-GPH method for the determination of collagen via the tripeptide Gly-Pro-Hyp, as column selection was crucial for this method, since amino acids and short peptides are polar and ionized substances and are therefore hardly retained. In such cases, the use of hydrophilic-interaction chromatographic columns may be preferred. Although three different stationary phases, different pH values (4.0, 6.0) of the mobile phase (ammonium formate buffer), and different amounts of organic modifier (70–85%) were tested, the method development was not successful, due to high noise, insufficient separation, or an inappropriate shape of the chromatographic peaks.

**Figure 2:**
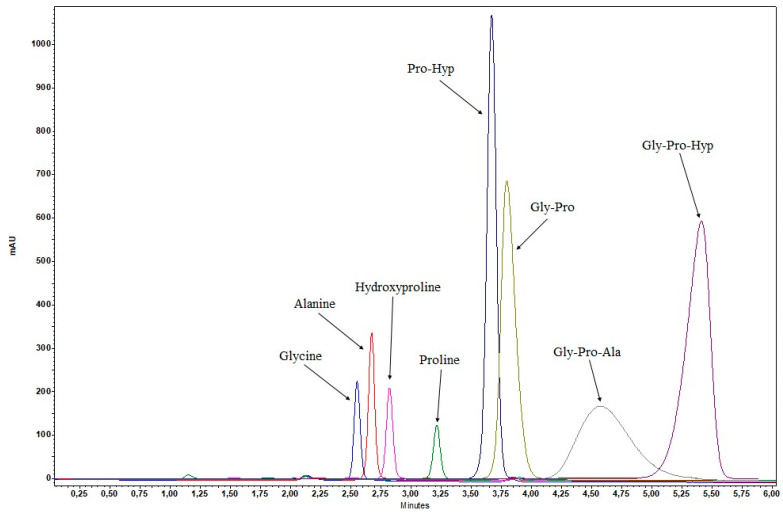
Selectivity of the HPLC-GPH method with respect to the separation of Gly-Pro-Hyp from other common collagen peptides (Pro-Hyp, Gly-Pro, and Gly-Pro-Ala) and amino acids (glycine, alanine, hydroxyproline, and proline).

Therefore, the development of the HPLC-GPH method was continued with reversed-phase columns. Numerous columns with different stationary phases (C18, C12, C3, F5) and various mobile phase additives (formic acid, trifluoroacetic acid, H_3_PO_4_, phosphate buffers) were tested. When formic acid was used, the analyte Gly-Pro-Hyp was not detected. With trifluoroacetic acid, the retention times were long, resulting in broad and asymmetric peaks. The most suitable peak shape was obtained with 0.1% H_3_PO_4_. Different column dimensions (50–250 mm) were tested, and separation was better with longer columns (250 × 4.6 mm). The best separation of Gly-Pro-Hyp from other common collagen peptides and amino acids was achieved with the Gemini C18 column (Figure 2). Another reason for choosing the Gemini C18 column was its stability under 100% aqueous conditions, which were found to be required due to the polarity of the analytes. Therefore, the combination of Gemini C18 column and 0.1% H_3_PO_4_ was applied to all developed RP-HPLC methods. To obtain the final HPLC-GPH method, the initial isocratic method under 100% aqueous conditions was extended by a gradient program with acetonitrile (ACN) to elute the more lipophilic sample components originating from collagen hydrolysate food supplements (larger peptides, other active ingredients, excipients, impurities) and sample preparation (enzyme).

**Figure 3:**
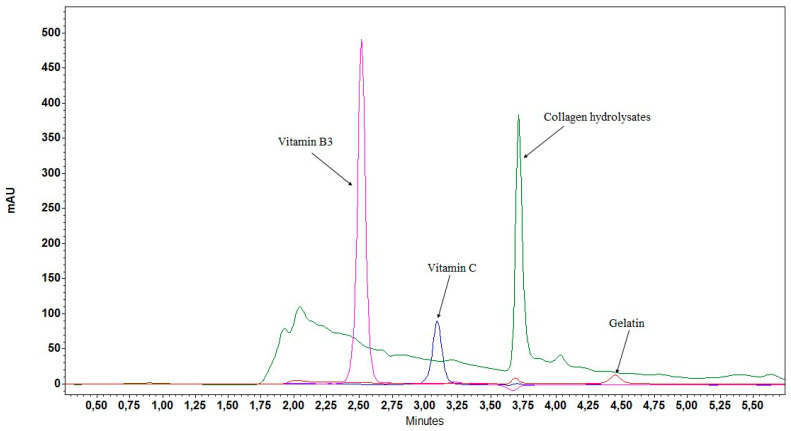
Characteristic chromatographic peak of collagen hydrolysates and selectivity of the HPLC-CH method.

After the development of the HPLC-GPH method for enzymatically hydrolyzed collagen samples, the development was continued with the method for untreated samples. No HPLC methods with UV detection were found in the literature that would provide a fingerprint chromatogram for the characterization of collagens of different origin. Despite many tested gradient programs, columns, and samples, we did not succeed in developing such a method. Therefore, a different direction was taken in the method development, and our aim was to elute all collagen hydrolysate peptides in a characteristic chromatographic peak (Figure 3). At a low percentage of ACN in the mobile phase (5%) sensitivity was low, while at a higher percentage of ACN (12%), separation was insufficient and selectivity was impaired. Therefore, an isocratic elution with 10% ACN was used in the final HPLC-CH method. This method allows a simple and quantitative evaluation of the collagen content.

Lastly, an attempt was made to improve the literature methods UV-dHyp and FLD-dNGP by chromatographic separation. Since the derivatized products are more lipophilic, a higher percentage of organic modifier in the mobile phase was required. Different proportions of ACN (30–50%) were tested in the HPLC-dHyp method development. The most suitable chromatographic peak shape was obtained at 30% ACN. Using the HPLC-dNGP method, we attempted to separate individual derivatized NGPs. Different proportions of organic modifiers (10–50%), different gradient programs, and the replacement of ACN with methanol were tested, but separation was not possible, probably due to the similar physicochemical properties of the derivatized NGPs. In terms of chromatographic peak shape, ACN performed better than methanol, while sensitivity was improved, with a higher percentage of ACN in the mobile phase. Therefore, an isocratic elution with 50% ACN was used in the final HPLC-dNGP method.

### 2.2. Method Validation

#### 2.2.1. Specificity

Collagen supplements often contain vitamins, minerals, plant extracts, or other natural products (Section 4.1). Assuming that minerals would not interfere with the developed methods, specificity was evaluated with solutions of coenzyme Q10 and vitamins A, B3, B7, and C. Vitamin A and coenzyme Q10 were not problematic for any of the methods due to their lipophilic nature, resulting in lower water solubility. For the other vitamins, the signal intensities were similar to the blank samples (spectroscopic methods), and no interfering peaks were observed at the retention times of our analytes (chromatographic methods). Vitamin C was an exception in the UV-BCA and HPLC-GPH methods. In the latter, the resolution between vitamin C and Gly-Pro-Hyp was only 0.4. Nevertheless, this did not affect the analysis, as vitamin C was present in the samples in very low concentrations (0–0.8 mg/L in analyzed samples) and was also degraded to a large extent due to its instability during sample preparation. The interaction was to be expected with the UV-BCA method, as vitamin C causes the reduction of Cu^2+^ ions and thus an apparently higher signal. The low specificity of the UV-BCA method is also evident in the analysis of collagen supplements, as very high collagen contents were determined (Section 2.3.2). This may be due to the presence of vitamin C or other active ingredients, excipients, or impurities (e.g., product B, which does not contain vitamin C).

The HPLC-GPH method is specific for the peptide Gly-Pro-Hyp, whereas the FLD-dNGP and HPLC-dNGP methods also detect other NGPs (e.g., Gly-Pro-Ala, Gly-Pro) and glycine. The use of chromatography did not improve specificity and did not prove to be of added value in this case. As expected, peptides that do not contain N-terminal glycine, such as the dipeptide Pro-Hyp, were not detected.

In the case of the HPLC-CH method, its specificity was additionally evaluated by analyzing native collagen and gelatin. No interfering peaks were observed (Figure 3). Due to the principle of the FLD-OPA method, the presence of primary amines (e.g., amino acids, Tris-HCl buffer, peptide and protein impurities, etc.) may interfere with quantification, causing apparently higher protein concentrations. Since the sensitivity of the FLD-OPA method depends on the number of primary amino groups (N-terminal amino group, lysine amino acid residue), it is very important that the degree of hydrolysis of the standard is similar to that of the samples, which was verified by the HPLC-SEC analysis. In addition, the FLD-OPA and UV-BCA methods are not specific for collagen. Therefore, any protein or peptide would be detected in the samples and cause a false high result. The HPLC-dHyp and UV-dHyp methods, on the other hand, are specific for collagen, as Hyp is very rarely found in other proteins [24].

#### 2.2.2. Linearity and Sensitivity

The calibration standards were fitted to a linear regression model. The only exception was the UV-BCA method, where a certain curvature of the data was observed. Therefore, the quadratic regression model was used for the UV-BCA method, which gave a better fit than the linear regression model. The use of the quadratic regression model is also recommended in the user manual of the BCA™ Protein Assay Kit [37]. The determination coefficient (R^2^) was higher than specified (R^2^ > 0.99) for all tested methods and on all three validation days. The two methods with the highest R^2^ values (R^2^ > 0.999) were the HPLC-GPH method and the HPLC-CH method for the marine collagen hydrolysate standard. The average R^2^ values are listed in Table 1.

According to the limit of quantification (LOQ) converted to the corresponding collagen concentrations (Table 1), the most sensitive methods are those based on fluorometric detection, namely the FLD-OPA method (3 mg/L), and the methods based on derivatized NGPs, i.e., the HPLC-dNGP method (4 mg/L) and the FLD-dNGP method (6 mg/L). Moderately sensitive are the HPLC-GPH method (16 mg/L) and the UV-BCA method (27 mg/L), followed by the methods based on Hyp determination (100 mg/L). The least sensitive is the HPLC-CH method (125 mg/L).

#### 2.2.3. Accuracy, Precision, and Recovery

The results of intra- and inter-day accuracy and precision are summarized in Table 1. The results for all methods are within the specified criteria for both intra-day accuracy and precision as well as for inter-day accuracy. However, the inter-day precision of the FLD-dNGP method deviates slightly from the set limit, while the relative standard deviation (RSD) of the FLD-OPA method is unacceptable. We found that quality control (QC) samples or calibration samples have to be analyzed together with the samples for each microplate.

To confirm the suitability of the developed methods for quantification purposes, the accuracy was additionally evaluated on commercially available collagen products as a means of method recovery determination (Section 2.3.2). The average recoveries for the FLD-OPA and UV-BCA methods were slightly below the lower acceptance limit (90%), while the other methods met the specified criteria.

#### 2.2.4. Sample Stability

The sample stability results are summarized in Table 1. For the FLD-OPA and UV-BCA methods, samples have to be measured immediately after preparation (<10 min) as they are the least stable. For the FLD-OPA method, the fluorescence intensity decreases with time, whereas for the UV-BCA method, the absorbance increases with time as the color continues to develop, since the UV-BCA method is not a true end-point method [37].

The QC samples for the HPLC-dNGP method were stable for only 1 h despite cooling the autosampler to 8 °C. Therefore, only a limited number of samples could be derivatized simultaneously in order to be analyzed in 1 h. The use of dark vials also proved to be advantageous for these samples, as the light-protected samples were slightly more stable than the unprotected ones. The derivatized QC samples for the FLD-dNGP method were stable for 4 h at room temperature. For the UV-dHyp and HPLC-dHyp methods, the derivatized QC samples were stable for 4 and 5 h at room temperature, respectively. Additionally, it was verified that the samples could be stored at room temperature for 24 h after basic hydrolysis and subsequent neutralization, allowing us to perform this time-consuming step one day in advance.

The QC samples for the HPLC-GPH and HPLC-CH methods are underivatized and therefore the most stable. They are stable for at least 96 h at room temperature.

### 2.3. Quality Control of Food Supplements

#### 2.3.1. Determination of Gly-Pro-Hyp and Hyp in Collagen

In order to evaluate the collagen content in food supplements, the percentage of Hyp or the tripeptide Gly-Pro-Hyp and other NGPs in collagen had to be determined for the methods based on the basic or enzymatic hydrolysis of collagen. For bovine collagen, the literature value of 12.5% of Hyp was used for the calculations [21]. The percentage of Hyp in marine collagen was determined to be 11.8% and 11.1% using the UV-dHyp and HPLC-dHyp methods, respectively. The results obtained with both methods are similar and the Hyp content is lower in marine collagen than in bovine collagen, which is consistent with the literature [7].

The results obtained with the HPLC-GPH method show that bovine and marine collagen contain 4.6% and 3.8% of the tripeptide Gly-Pro-Hyp, respectively. Since the percentage of Hyp in marine collagen is lower, it was expected that the percentage of Gly-Pro-Hyp in marine collagen would also be lower. The difference is considerable and makes it possible to distinguish between marine and terrestrial collagen using this method.

The FLD-dNGP and HPLC-dNGP methods are less specific, since in addition to Gly-Pro-Hyp, all NGPs are derivatized with 3,4-dihydroxyphenylacetic acid. Using the HPLC-dNGP method, bovine and marine collagen were found to contain 31.3% and 29.8% NGPs, respectively. The values did not differ considerably between bovine and marine collagen. For the FLD-dNGP method, similar values were determined for bovine and marine collagen, namely 20.0% and 20.2%, respectively. Therefore, it is not possible to distinguish between collagen of different origins using the HPLC-dNGP and FLD-dNGP methods.

#### 2.3.2. Quality Control of Commercially Available Products

Ten commercially available food supplements in different dosage forms (powders, capsules, oral solutions) containing hydrolyzed collagen of various origins were analyzed using all the aforementioned methods. The results are shown in Table 2.

The HPLC-SEC method was used to evaluate the size distribution of the peptides in the products. All products contained hydrolyzed collagen with a similar size distribution of peptides. However, product A had a wider peptide size distribution and contained slightly larger peptides. This was also reflected in the collagen content determined by the FLD-OPA method (70.8%). Since the sensitivity of the FLD-OPA method depends on the number of primary amino groups, the collagen content determined by this method was falsely lower in this sample. Although, the peptide size distribution for product C was the same as for the other products, a relatively low collagen content (70.3%) was determined with the FLD-OPA method, but not with the other methods, indicating that there may be interference with other active ingredients or excipients.

The low specificity of the UV-BCA method was already confirmed in the method validation (Section 2.2.1). As a result, falsely high collagen contents were determined in food supplements. This is due to the presence of vitamin C, but it can also be caused by other active ingredients, excipients, or impurities.

The collagen content in the food supplements was mostly around 100%. No trends were identified with regard to the influence of the dosage form or the origin of the collagen on the content. In general, the lowest contents were determined using the FLD-dNGP method (77.4–95.2%). The largest range of values (87.5–117.9%) was obtained using the UV-dHyp method, probably due to poor repeatability. The collagen contents determined with different methods (with the exception of the UV-BCA method) for the same product are comparable, as the results are mostly within ±10%. The largest variation in the results obtained with the different methods was observed for product H (77.4–112.9%).

In general, the repeatability was better when using chromatographic methods than when using spectroscopic methods. As expected, the lowest RSD values were obtained with the HPLC-CH method (average RSD 2.2%), as the sample preparation is the simplest. The HPLC-GPH method, in which the samples are enzymatically hydrolyzed and no further sample preparation is required, had similar repeatability (average RSD 2.3%). The repeatability was lowest for the methods with more complex sample preparation (hydrolysis and derivatization), especially for the UV-dHyp method (average RSD 9.7%), but still within the specified limits (RSD < 10%).

The HPLC-GPH method can, with high probability, distinguish between marine and terrestrial collagen, as the Gly-Pro-Hyp content in collagens of different origins varies. Therefore, we investigated whether the origin of the collagen matched the labeled origin (Table 3). For most products, the labeled origin matched the identified origin, as terrestrial collagen contains 4.6% ± 0.2% and marine collagen contains less than 4.0% of the tripeptide Gly-Pro-Hyp. An exception was product E, which contains marine, bovine, and chicken collagen, with marine collagen accounting for about one third of the total collagen. From the determined Gly-Pro-Hyp content (4.62%), it can be concluded that this product most likely does not contain marine collagen, as the Gly-Pro-Hyp content is too high. Based on the Gly-Pro-Hyp contents in the marine and bovine collagen standards, we would expect Gly-Pro-Hyp content of about 4.3% in this product. Product H was the only one where the origin of the collagen was not labeled. Using the HPLC-GPH method, we assume that product H did not contain marine but terrestrial collagen.

### 2.4. Comparison of Analytical Methods

The developed and established methods were compared with respect to key parameters that appropriately evaluate the performance of the methods: specificity, sample stability and other validation parameters, complexity of sample preparation, and suitability for the quality control of collagen-containing food supplements (Table 4).

One of the most important parameters is specificity, which ensures that the analysis is not affected by other active substances, excipients, or impurities. The least suitable methods in terms of specificity are the UV-BCA and FLD-OPA methods, as they are not specific for collagen and the results are affected by other substances contained in collagen supplements or the degree of collagen hydrolysis (Section 2.2.1). The HPLC-CH method does not separate the individual peptides in the collagen hydrolysate, and no interferences from common ingredients of collagen supplements and native or partially hydrolyzed collagen (e.g., gelatin) were observed. The HPLC-dHyp and UV-dHyp methods are relatively specific for collagen, as Hyp is very rare in other proteins [24]. The FLD-dNGP and HPLC-dNGP methods are relatively specific for collagen, as the signal intensities for other proteins are insignificant [22]. However, N-terminal glycine-containing dipeptides (e.g., Gly-Pro) and glycine itself also generate a measurable signal. The tripeptide Gly-Pro-Hyp can be specifically determined using the HPLC-GPH method, which is also the only method that can distinguish between marine and terrestrial collagen. Therefore, it was classified as the best in terms of specificity.

When considering other validation parameters, the FLD-OPA method and the FLD-dNGP method performed the worst due to insufficient inter-day precision. The other methods met the specified validation criteria. The samples prepared for analysis with the HPLC-CH and HPLC-GPH methods are most stable. The derivatized samples are less stable and therefore have to be analyzed soon after preparation, especially when using the UV-BCA and FLD-OPA methods. Due to the duration of the analysis, the samples for the HPLC-dNGP method have to be prepared in batches, which is time-consuming and inefficient.

Sample preparation is simplest with the HPLC-CH method, in which the samples are simply dissolved in water (capsules, powders) or diluted with water (oral solutions). This is followed by the FLD-OPA and UV-BCA methods, in which the samples are quickly derivatized, and the HPLC-GPH method, in which the samples are hydrolyzed within one hour. The most time-consuming methods are those in which both hydrolysis and derivatization are carried out, as many steps and the daily preparation of many reagents are required. Methods involving basic hydrolysis are particularly complex and time-consuming due to the autoclaving and the two derivatization steps. Sample preparation therefore takes at least three times as long as for methods involving enzymatic hydrolysis. In addition, the HPLC-dHyp and UV-dHyp methods are less suitable for routine use from a safety point of view, as strong acids (3.5 M H_2_SO_4_, 60% perchloric acid) and bases (7 M NaOH) are used in sample preparation. The analysis itself is generally faster with spectroscopic methods than with chromatographic methods; however, the samples can be analyzed automatically with HPLC systems.

When analyzing the food supplements, the HPLC-GPH method proved to be the best, as it is the only method that can differentiate between marine and terrestrial collagen (Table 3). The UV-BCA and FLD-OPA methods are inadequate due to their low specificity, and the UV-dHyp method is also less suitable due to its poor repeatability.

Among all the parameters evaluated, the HPLC-CH and HPLC-GPH methods proved to be best suited, mainly due to its specificity, stability of samples, and simplicity of sample preparation. The HPLC-GPH method stands out in particular, as it is the only one that distinguishes between marine and terrestrial collagen, which is a novelty in the field of simple HPLC-UV methods for collagen analysis.

## 3. Discussion

Maintaining a youthful appearance, concealing signs of aging, and other beauty trends are important values in modern society, which is why consumers often turn to anti-aging products. Food supplements are widely available and are often consumed over long periods of time and are perceived as safe by consumers due to their similarity to pharmaceuticals. On the other hand, there is a lack of legislation and regulatory controls in this area, resulting in substandard products being placed on the market [9,10,11,12,13,14,15]. A literature review revealed a lack of data on the quality of food supplements containing collagen, a popular anti-aging nutricosmetic ingredient. Therefore, the development of appropriate analytical methods for the quality control of collagen-containing products is important to ensure their efficacy and safety, with the content, degree of hydrolysis, and origin of the collagen being of particular importance.

Currently, the analysis of collagen using chromatographic techniques is still based on coupling with a mass spectrometer [23,31,32,33] or, similar to spectroscopic methods, on the determination of derivatized Hyp [27,28,29]. The main objective of our work was therefore to develop simple HPLC-UV methods for the quantitative determination of collagen content in food supplements without the need for derivatization (Figure 1). Since the origin of collagen affects safety, the ability of the methods to distinguish marine from terrestrial collagen was of utmost importance. Two established literature methods were adapted as reference methods, namely the spectroscopic method for the determination of derivatized Hyp after the basic hydrolysis of collagen (UV-dHyp) [21] and the method with the fluorescence detection of derivatized NGPs after the enzymatic hydrolysis of collagen with bacterial collagenase (FLD-dNGP) [22]. We investigated whether the specificity of these two methods could be improved by chromatographic separation (HPLC-dNGP and HPLC-dHyp). The next objective was to develop a method for the determination of collagen via the underivatized tripeptide Gly-Pro-Hyp (HPLC-GPH), which is the most abundant tripeptide formed during the enzymatic hydrolysis of collagen. The third concept was the development of an HPLC method for the analysis of untreated samples of food supplements containing collagen hydrolysates (HPLC-CH). Furthermore, the possibility of using simple and established techniques for the evaluation of protein concentration was explored.

First, two new methods were introduced, namely the HPLC-CH method, which enables the analysis of collagen hydrolysates in untreated samples of food supplements, and the HPLC-GPH method for the determination of underivatized tripeptide Gly-Pro-Hyp, which is produced during the enzymatic hydrolysis of collagen. Both methods represent a major advantage in the field of collagen analysis, as they are simple and fast, and do not require expensive mass spectrometers or time-consuming sample derivatization. The latter is not only advantageous in terms of the simplicity of sample preparation, but also in terms of sample stability, as non-derivatized samples are much more stable than derivatized samples (Table 1). Both methods also proved to be superior in terms of specificity and other validation parameters. In addition, both methods use the same combination of chromatographic column and mobile phase, so they can be used sequentially.

The greatest advantage of the HPLC-CH method is the speed of analysis (9 min) and the simplicity of sample preparation, as no special treatment is required. The tripeptide Gly-Pro-Hyp is specifically determined using the HPLC-GPH method. The biggest advantage of this method is therefore its specificity, which is even better than that of methods based on the determination of Hyp or NGPs. Collagen is one of the few proteins containing Hyp, so the probability that the above-mentioned tripeptide is formed during the hydrolysis of other proteins or peptides by bacterial collagenase is practically zero. In addition, the HPLC-GPH method is the only method that can distinguish between marine and terrestrial collagen with a high degree of certainty, as the content of the Gly-Pro-Hyp tripeptide varies between collagens of different origin [30]. Compared to terrestrial collagens, marine collagens have lower denaturation temperatures because they contain less Hyp, which is important for the stabilization of the triple helix [7]. A difference in Hyp content was also observed in our experiments using the UV-dHyp and HPLC-dHyp methods, but it was too small and the sample preparation repeatability was also not optimal, so it is not possible to distinguish between the collagens of different origin using these two methods. However, we emphasize that differentiation using the HPLC-GPH method is only possible when it is used in conjunction with a method that determines the collagen concentration in the sample regardless of its origin (e.g., using the HPLC-CH method), so that the proportion of the tripeptide Gly-Pro-Hyp can be calculated and then the marine or terrestrial origin can be determined. This case also demonstrates the importance of the simultaneous use of complementary analytical techniques in protein analysis. The only issue with the HPLC-GPH method is the somewhat longer duration of the analysis (23 min), which is due to the gradient elution. The method could be shortened and optimized in the future. However, as the samples are stable at least for several days in the autosampler at room temperature and HPLC systems enable automated analysis, this disadvantage is negligible.

We found that the established literature methods have several drawbacks compared to our newly developed methods, mainly due to the complexity of sample preparation, sample instability, and non-selectivity. We investigated whether these methods could be improved by chromatography. Although chromatographic methods for the determination of collagen via derivatized Hyp can be found in the literature [27,28,29], we believe that the addition of chromatographic separation does not offer any advantages over spectroscopic methods. We have found that the HPLC-dHyp and HPLC-dNGP methods developed do not add value compared to the spectroscopic UV-dHyp and FLD-dNGP methods, as spectrophotometric analysis is cheaper and faster, the instability of derivatized samples is therefore less of a problem, and, crucially, selectivity is not improved with the HPLC techniques.

Of the methods tested, the FLD-OPA and UV-BCA methods, which are generally used to determine the total protein concentration in samples, proved to be the worst in terms of validation parameters and suitability for the quality control of collagen-containing food supplements. The main disadvantage of these methods is their non-specificity, which means that any peptide or protein can be detected in the samples. With the FLD-OPA method, the degree of collagen hydrolysis also affects the analysis result, whereas with the UV-BCA method, other ingredients of the food supplements interfere with the analysis. The low specificity of the UV-BCA method and possible interferences with the ingredients of food supplements were also observed when analyzing food supplements containing other proteins [38].

The developed methods have been used for the quality control of commercially available collagen products, which is important for achieving anti-aging effects and for consumer protection. The regulations for food supplements are less strict than those for pharmaceuticals. According to the European Commission, the vitamin content is considered adequate if it is between 80% and 150% [39], and such values were therefore expected for collagen supplements. We found that the collagen content was adequate in all products tested, as the values were close to 100%. In some anti-aging products, the instability of the active ingredients results in lower content [15,16,17,18,19], which is apparently not the case with collagen (Table 2). Although the active ingredients in liquid dosage forms (products F, G, and I) are generally less stable than in solid dosage forms, the collagen content was sufficient in all products tested. In addition, some anti-aging products could be subject to adulteration, as active ingredients are missing or replaced by cheaper and less effective alternatives [12]. This was also not the case with collagen-containing supplements, as it is a relatively cheap ingredient that can be easily obtained from by-products of the food industry. Using the HPLC-SEC method, we have confirmed that all tested products contain low-molecular-weight peptides, which have better bioactivity than larger collagen polypeptide chains [8].

In terms of safety, it is important to distinguish between marine and terrestrial collagen. Since the content of tripeptide Gly-Pro-Hyp varies in collagens of different origins, the HPLC-GPH method can be used to routinely and with high probability determine whether the food supplement contains marine or terrestrial collagen (Table 3). For most products, we have confirmed that they contain collagen of the specified origin. Product E was supposed to contain a mixture of marine and terrestrial collagen; however, it was found that it most likely did not contain marine collagen, as the Gly-Pro-Hyp content was too high. The only product where the origin of the collagen was not specified (product H) was found to most likely contain terrestrial collagen. Appropriate labeling and origin control are important to consumers for religious reasons. In addition, the origin of the collagen has safety implications, as terrestrial collagen has been associated with the transmission of various diseases and the risk of triggering an immune response (in about 3% of the population) [6]. On the other hand, marine collagen can trigger an allergic reaction in people allergic to seafood [40].

In conclusion, our work shows how important the quality control of anti-aging products, such as collagen supplements, is for their efficacy and safety, whereby the development of appropriate analytical methods plays an important role. To this end, two new HPLC-UV methods have been developed, one of which is characterized by its speed and simplicity of sample preparation, and the other by its specificity and ability to distinguish between marine and terrestrial collagen, which is particularly important in terms of safety. The results of our collagen analysis in commercially available products have confirmed that the proposed methods are suitable for routine analysis as they are simple, specific, and do not require specific skills and expensive equipment (mass spectrometers). A comparison of the developed chromatographic methods and established spectroscopic reference methods undoubtedly shows that the HPLC-CH and HPLC-GPH methods are best suited for the quality control of collagen-containing food supplements. In the future, it would be useful to investigate the applicability of the developed methods for the quality control of collagen-containing cosmetic products, which are also advertised as having anti-aging effects, as it has been shown that the quality of cosmetic products is often questionable.

## 4. Materials and Methods

### 4.1. Materials

The peptides Gly-Pro-Hyp, Gly-Pro, Pro-Hyp, and Gly-Pro-Ala were purchased from Bachem (Bubendorf, Switzerland). Marine collagen hydrolysate standard (100%) and bovine collagen hydrolysate standard (99.8%) were purchased from Valens d.o.o (Komenda, Slovenia) and Medex d.o.o. (Ljubljana, Slovenia), respectively. Collagen A (native bovine collagen standard; 1.3 mg/mL), trans-4-hydroxy-L-proline (≥99%) 3,4-dihydroxyphenylacetic acid, 4-(dimethlyamino)benzaldehyde, acetic acid, ammonium formate, coenzyme Q10, collagenase from *Clostridium histolyticum*, chloramine T trihydrate, L-alanine, L-proline, perchloric acid (60%), potassium dihydrogen phosphate, sodium hydroxide, sulfuric acid, and trifluoroacetic acid were purchased from Sigma-Aldrich (St. Louis, MO, USA). HPLC-grade ACN, HPLC-grade isopropanol, HPLC-grade methanol, ethanol (96% p.a.), and sodium periodate were purchased from Honeywell (Charlotte, NC, USA). Boric acid, calcium chloride dihydrate, citric acid monohydrate, food-grade gelatin, formic acid, glycine, hydrochloric acid for a 1 M solution (Titrisol^®^), 2-mercaptoethanol, ortho-phosphoric acid, OPA, sodium acetate trihydrate, sodium hydroxide for a 1 M solution (Titrisol^®^), and tris(hydroxymethyl)aminomethane were purchased from Merck (Darmstadt, Germany). L-ascorbic acid, niacinamide, and vitamin A acetate were purchased from Biosynth (Staad, Switzerland). Biotin was purchased from Carbosynth (Berkshire, UK). The BCA™ Protein Assay Kit was purchased from Thermo Scientific (Rockford, IL, USA). Minisart^®^ filters (0.20 μm) were purchased from Sartorius (Göttingen, Germany). High-purity Milli-Q^®^ water was obtained using a Milli-Q^®^ A10 Advantage water purification system (Millipore Corporation, Bedford, MA, USA). Ten commercially available food supplements containing hydrolyzed collagen were purchased from a Slovenian market. Additional data on the samples can be found in Table 5.

Several chromatographic columns were tested during the method development. Acquity UPLC^®^ HSS T3 (50 × 2.1 mm, 1.8 µm) and XSelect CSH C18 (150 × 4.6 mm, 3.5 µm) were purchased from Waters Corporation (Milford, MA, USA). BioSep-SEC-S2000 (300 × 7.8 mm, 5 µm), Gemini C18 (50 × 4.6 mm, 5 µm; and 250 × 4.6 mm, 5 µm), Kinetex F5 (100 × 4.6 mm, 2.6 µm), Kinetex HILIC (100 × 2.1 mm, 2.6 µm), Luna C18(2) (250 × 4.6 mm, 5 µm; and 250 × 4.6 mm, 3 µm), Luna Omega Sugar (150 × 4.6 mm, 3 µm), Synergy Hydro RP (150 × 4.6 mm, 4 µm; and 250 × 4.6 mm, 4 µm), and Synergy MAX-RP (150 × 4.6 mm, 4 µm) were purchased from Phenomenex (Torrance, CA, USA). ZIC-HILIC (50 × 2.1 mm, 3.5 µm) was purchased from Merck (Darmstadt, Germany). Zorbax 300SB-C3 (150 × 2.1 mm, 5 µm) was purchased from Agilent Technologies (Santa Clara, CA, USA).

### 4.2. Analytical Methods

#### 4.2.1. Sample Hydrolysis and Derivatization

For all methods, the collagen samples were dissolved in Milli-Q^®^ water. For the HPLC-CH method, these samples were only filtered and then analyzed. For the HPLC-GPH, HPLC-dNGP, and FLD-dNGP methods, enzymatic hydrolysis of the collagen was performed. The enzymatic hydrolysis and subsequent derivatization of the NGPs was carried out according to a slightly modified procedure as described by Yasmin et al. [22]. First, 260 μL of 125 mM sodium borate buffer (pH 8.0) and 40 μL of collagenase solution (0.1 mg/mL) in 50 mM Tris buffer containing 5 mM CaCl_2_ (pH 7.5) were added to 200 μL of collagen sample solution. The sample was mixed with a vortex mixer Vibromix 10 (Tehtnica, Železniki, Slovenia) and then incubated in a HeatMix (Tehtnica, Železniki, Slovenia) for 1 h at 37 °C with gentle shaking (20 rpm). For the HPLC-GPH method, half of the sample was transferred to vials and analyzed. For the HPLC-dNGP and FLD-dNGP methods, the second half of the sample was derivatized according to the following procedure: 250 μL of 1.25 mM 3,4-dihydroxyphenylacetic acid in Milli-Q^®^ water, 250 μL of 125 mM sodium borate buffer (pH 8.0), and 250 μL of 2.0 mM NaIO_4_ in Milli-Q^®^ water were added, mixed with a Vibromix 10, and incubated in a HeatMix at 37 °C for 10 min with gentle shaking (20 rpm). The reagent solutions were freshly prepared each day and protected from direct light. The samples were filtered and analyzed.

For the HPLC-dHyp and UV-dHyp methods, the basic hydrolysis of the collagen and the subsequent derivatization of Hyp were carried out according to a slightly modified procedure as described by da Silva et al. [21]: 800 μL of the collagen sample was pipetted into a 5 mL flask and 2.0 mL of 7.0 M NaOH was added. The prepared sample was autoclaved in autoclave A-21 (Kambič, Semič, Slovenia) for 70 min at a temperature of 121 °C and an overpressure of 1 bar. The samples were then cooled on ice, and 2.2 mL of 3.5 M H_2_SO_4_ was added. A total of 400 μL of the sample was transferred to a microcentrifuge, and 600 μL of 0.056 M chloramine T in acetate-citrate buffer (pH 6.5) was added. The sample was mixed with a Vibromix 10. The reaction then took place for 20 min at room temperature. Subsequently, 1.0 mL of Ehrlich’s reagent (1.0 M 4-dimethylaminobenzaldehyde in isopropanol:HClO_4_ = 2:1) was added, mixed again, and incubated in a HeatMix at 65 °C for 15 min. The reagent solutions were freshly prepared each day and protected from direct light. The sample was cooled to room temperature and filtered.

For the derivatization of collagen with OPA, 6 mg of OPA was dissolved in 100 μL of ethanol. A total of 20 μL of 2-mercaptoethanol was added and 50 mM borate buffer (pH 10.0) was made up to 10 mL. The reagent solution was freshly prepared each day and protected from direct light. A total of 50 μL of the reagent solution was added to 200 μL of the sample, shaken for 15 s, and measured immediately.

The collagen content was also evaluated by the UV-BCA method using the BCA™ Protein Assay Kit. The samples were prepared as described in the user manual (sample-to-working reagent ratio = 1:8) [37].

#### 4.2.2. Spectroscopic Methods

For the UV-dHyp method, the absorbance of the samples after basic hydrolysis and derivatization was measured at 562 nm using an Agilent 8453 UV-VIS spectrophotometer (Agilent Technologies, Santa Clara, CA, USA).

A Safire^2^ microplate reader (Tecan, Männedorf, Switzerland) was used for microplate measurements. For the FLD-dNGP method, 250 μL of the sample was transferred to black Cellstar^®^ 96-well microplates (Greiner Bio One, Kremsmünster, Austria) and the fluorescence intensity was measured. The excitation and emission wavelengths were set to 375 nm and 465 nm, respectively. Both slits were set to 9 nm. For the FLD-OPA method, kinetic fluorescence measurements (every 30 s, 21 cycles) were performed on black Cellstar^®^ 96-well microplates (Greiner Bio-One, Kremsmünster, Austria). The excitation and emission wavelengths were set to 340 nm and 440 nm, respectively. Both slits were set to 9 nm. The time point at which the majority of the samples on the same microplate showed the maximum fluorescence intensity was used for the calculations. For the UV-BCA method, absorbance measurements were carried out at 562 nm. White 96-well microplates with a transparent flat bottom were used for the measurements (Greiner Bio-One, Kremsmünster, Austria).

#### 4.2.3. RP-HPLC Methods

The RP-HPLC analyses were performed on an Agilent 1290 Infinity series UHPLC system (Agilent Technologies, Santa Clara, CA, USA), equipped with a diode array detector, a fluorescence detector, and the EZChrom (A.04.05) data acquisition program. Separations were performed on a Gemini C18 column (250 × 4.6 mm, 5 μm; Phenomenex, Torrance, CA, USA) at 30 °C via isocratic or gradient elution using 0.1% H_3_PO_4_ in Milli-Q^®^ water (A) and ACN (B) as the mobile phase at a flow rate of 1.0 mL/min. The injection volume was 10 μL. These chromatographic conditions were used for all RP-HPLC methods. For the HPLC-GPH method, the following gradient program was used: 0.0–10.0 min 0% B; 10.0–15.0 min 0–35% B; 15.0–18.0 min 35% B; 18.0–18.1 min 35–0% B; 18.1–23.0 min 0% B. UV detection was carried out at 214 nm. For the HPLC-CH method, an isocratic elution with a mobile phase ratio of A:B = 90:10 was used. UV detection was carried out at 214 nm. For the HPLC-dHyp method, an isocratic elution with a mobile phase ratio of A:B = 70:30 was used. UV detection was carried out at 562 nm. For the HPLC-dNGP method, an isocratic elution with a mobile phase ratio of A:B = 50:50 was used. The excitation and emission wavelengths for the fluorometric detection were set to 375 nm and 465 nm, respectively. Both slits were set to 9 nm.

#### 4.2.4. HPLC-SEC Method

An Agilent 1100 series HPLC system (Agilent Technologies, Santa Clara, CA, USA), equipped with a diode array detector and the ChemStation data acquisition program was used. Detection was carried out at 214 nm. Separation was performed on a BioSep-SEC-S2000 column (300 × 7.8 mm, 5 μm, Phenomenex, Torrance, CA, USA) at 25 °C. Isocratic elution was performed using 0.1% trifluoroacetic acid in Milli-Q^®^ water (A) and ACN (B) as a mobile phase (A:B = 85:15) at a flow rate of 1.0 mL/min. The injection volume was 20 μL.

### 4.3. Method Validation

All spectroscopic and RP-HPLC methods were validated according to the ICH Q2 (R1) guidelines [41]. The specificity, linearity, limit of detection (LOD), LOQ, accuracy, precision, and sample stability were evaluated.

#### 4.3.1. Specificity

Specificity was evaluated by comparing the signal intensity (spectroscopic methods) or chromatograms (HPLC methods) of blank samples, native collagen, gelatin, collagen hydrolysates, common collagen di- and tripeptides, and the most common ingredients of collagen supplements (coenzyme Q10, vitamins A, B3, B7, and C). The chromatograms were visually inspected for interfering peaks at the retention time of our analyte.

#### 4.3.2. Calibration Curve

Depending on the method, 5–7 calibration standards were used for the calibration curve. The corresponding equations and R^2^ were determined based on the least-square regression. Only for the UV-BCA method, a quadratic equation was chosen as suggested in the user manual [37]. A linear equation was used for the other methods. The acceptance limit was set to R^2^ > 0.99.

#### 4.3.3. LOD and LOQ

LOD and LOQ were calculated according to the ICH Q2 (R1) guidelines [41] based on the standard deviation of the response and the slope. The LOD was calculated as 3.3 σ/S and the LOQ was calculated as 10 σ/S, where σ represents the standard deviation of the y-intercepts of the calibration curve and S represents the average slope of the calibration curves. For the FLD-OPA method, σ was calculated as the standard deviation of the blank samples (n = 4) due to poor inter-day repeatability (Table 1). For the UV-BCA method, σ was calculated as the standard deviation of the blank samples (n = 4) and S was calculated by linear regression of the first three points of the calibration curve due to the use of a quadratic calibration equation.

#### 4.3.4. Accuracy and Precision

The intra- and inter-day accuracy and precision of the RP-HPLC and spectroscopic methods were evaluated on three validation days. Six replicate analyses of QC samples were performed. Precision, calculated as RSD, should be less than 10%. Accuracy was defined as the ratio (%) between the determined and the theoretical concentration. It should be between 90 and 110%.

Method recovery was evaluated in three parallels by separately analyzing the spiked preparations, the non-spiked preparations, and the standard solutions containing the added amounts of the analyte. The average recoveries were calculated using Equation (1). The acceptance limit was set at 90–110%.

(1) $$Recovery\;\left[\%\right]=100\times \frac{\mathrm{concentration}\;\mathrm{in}\;\mathrm{spiked}\;\mathrm{sample}\;-\;\mathrm{concentration}\;\mathrm{in}\;\mathrm{non}-\mathrm{spiked}\;\mathrm{sample}\;}{\mathrm{added}\;\mathrm{concentration}}$$

#### 4.3.5. Sample Stability

Sample stability was also evaluated by re-analyzing the QC samples after a certain time (depending on the method). The analyte concentration at a certain time point was compared to that at time zero. Samples were considered stable if the ratio expressed in percent was between 90 and 110%.

### 4.4. Sample Preparation Protocols

#### 4.4.1. Specificity

Specificity was evaluated by analyzing 0.01 mg/mL aqueous solutions of coenzyme Q10 and vitamins A, B3, B7, and C, which are commonly contained in collagen supplements. Blank samples were also analyzed for all the methods using water instead of the analyte. For the HPLC-CH method, gelatin and native collagen at a concentration of 0.5 mg/mL were additionally analyzed. The specificity of the HPLC-GPH method was additionally evaluated using aqueous solutions of amino acids (1.0 mg/mL; glycine, alanine, Pro, and Hyp), dipeptides (0.1 mg/mL; Pro-Hyp, and Gly-Pro), and tripeptide (0.1 mg/mL; Gly-Pro-Ala). For the FLD-dNGP and HPLC-dNGP methods, the di-and tripeptide samples were diluted to 0.025 mg/L with Milli-Q^®^ water. To 100 μL of the sample, 130 μL of 125 mM sodium borate buffer (pH 8.0) and 20 μL of Tris buffer (pH 7.5) was added, followed by the derivatization procedure described in Section 4.2.1.

#### 4.4.2. Calibration Curve, Precision, and Accuracy

For all methods, the calibration samples were prepared in two parallels and the QC samples in six parallels at one concentration level.

For the HPLC-GPH, FLD-dNGP, and HPLC-dNGP methods, the Gly-Pro-Hyp stock solution (160 mg/L) was prepared by dissolving the Gly-Pro-Hyp standard in Milli-Q^®^ water. The stock solution was diluted with Milli-Q^®^ water to obtain six calibration samples with the following concentrations: 1, 2, 4, 8, 12, and 16 mg/L. For all three methods, the QC samples were prepared at a concentration of 8 mg/L by diluting the Gly-Pro-Hyp stock solution.

For the UV-dHyp and HPLC-dHyp methods, the Hyp stock solution (75.0 mg/L) was prepared by dissolving the Hyp standard in Milli-Q^®^ water. The stock solution was diluted with Milli-Q^®^ water to obtain seven calibration samples with the following concentrations: 6.25, 12.5, 25.0, 37.5, 50.0, 62.5, and 75.0 mg/L. For both methods, the QC samples were prepared at a concentration of 25.0 mg/L by diluting the Hyp stock solution.

For the HPLC-CH method, the stock solutions (4000 mg/L) were prepared by dissolving marine or bovine collagen hydrolysate standards in Milli-Q^®^ water. Each stock solution was diluted with Milli-Q^®^ water to obtain six calibration samples with the following concentrations: 125, 250, 500, 1000, 2000, and 4000 mg/L. The QC samples were prepared at a concentration of 500 mg/L by diluting the stock solution.

For the UV-BCA method, the stock solutions (2000 mg/L) were prepared by dissolving marine or bovine collagen hydrolysate standards in Milli-Q^®^ water. Each stock solution was diluted with Milli-Q^®^ water to obtain seven calibration samples with the following concentrations: 31.25, 62.5, 125, 250, 500, 1000, and 2000 mg/L. The QC samples were prepared at a concentration of 500 mg/L by diluting the stock solution.

For the FLD-OPA method, the stock solutions (100 mg/L) were prepared by dissolving marine or bovine collagen hydrolysate standards in Milli-Q^®^ water. Each stock solution was diluted with Milli-Q^®^ water to obtain five calibration samples with the following concentrations: 5, 10, 25, 50, and 75 mg/L. The QC samples were prepared at a concentration of 50 mg/L by diluting the stock solution.

#### 4.4.3. Determination of Gly-Pro-Hyp and Hyp in Collagen

The percentage of the tripeptide Gly-Pro-Hyp and other NGPs had to be determined for the HPLC-GPH, HPLC-dNGP, and FLD-dNGP methods in order to determine the collagen content. Aqueous solutions of marine collagen hydrolysate standard and native bovine collagen standard were prepared at a concentration of 50 mg/L. The sample preparation procedure was continued as described in Section 4.2.1. At least three parallels of each standard were analyzed. To determine the percentage of Gly-Pro-Hyp or NGPs in collagen, the concentration of the Gly-Pro-Hyp or NGPs was calculated using the corresponding regression line, divided by the collagen concentration in the collagen sample, and expressed as a percentage. The same procedure was used to determine the origin of the collagen in the products by determining the content of the tripeptide Gly-Pro-Hyp, considering the collagen concentration in the sample determined by the HPLC-CH method.

Similarly, for the HPLC-dHyp and UV-dHyp methods, the percentage of Hyp in the collagen was determined. An aqueous solution of marine collagen hydrolysate standard was prepared at a concentration of 250 mg/L. The sample preparation procedure was continued as described in Section 4.2.1. To determine the percentage of Hyp in collagen, the concentration of the Hyp was calculated using the regression line, divided by the collagen concentration in the collagen sample, and expressed as a percentage. For the bovine collagen, the literature value of 12.5% of Hyp was used for the calculations [21].

#### 4.4.4. Evaluation of Collagen Content in Commercially Available Products

Samples of commercially available products were prepared by dissolving the appropriate mass or volume (depending on the labeled content; see Table 5) of the product in Milli-Q^®^ water to obtain a collagen concentration of approximately 500 mg/L. For the UV-dHyp and HPLC-dHyp methods, the samples were diluted twice (250 mg/L), while for the FLD-OPA, FLD-dNGP, HPLC-dNGP, and HPLC-GPH methods, the samples were diluted ten times (50 mg/L) before the hydrolysis and/or derivatization procedures were performed. All samples were prepared in triplicate.

#### 4.4.5. Recovery

The method recovery was determined by spiking the products containing collagen hydrolysates with approximately the same amount of the standard, so that the concentration in the spiked sample was approximately equal to the concentration in the QC samples. All recovery samples were prepared in triplicate.

## Figures and Tables

**Table 1:** Validation data.

Method	Analyte		Validation Parameters						
		Range [g/L]	LOD/LOQ [g/L]	R^2^	Precision [%]		Accuracy [%]		Stability
					Intra-Day	Inter-Day	Intra-Day	Inter-Day	
HPLC-CH	MCH	0.125–4.000	0.023/0.070	0.9991	2.0	7.8	98.8	97.2	96 h at RT
	BCH		0.039/0.119	0.9980	3.3	5.8	96.7	96.1	
HPLC-GPH	GPH	0.001–0.016 (0.025–0.400) *	0.0002/0.0006	0.9995	3.1	1.0	98.3	97.9	96 h at RT
			(0.005/0.016) *						
HPLC-dNGP	GPH	0.002–0.016 (0.006–0.053) *	0.0006/0.0019	0.9978	2.7	5.6	101.9	98.2	1 h at 8 °C
			(0.002/0.006) *						
FLD-dNGP	GPH	0.001–0.016 (0.005–0.080) *	0.0002/0.0007	0.9985	2.5	10.3	107.5	100.4	4 h at RT
			(0.001/0.004) *						
HPLC-dHyp	Hyp	0.013–0.075 (0.100–0.600) *	0.004/0.013	0.9970	5.3	3.4	105.1	98.8	5 h at RT
			(0.034/0.103) *						
UV-dHyp	Hyp	0.013–0.075 (0.100–0.600) *	0.004/0.011	0.9970	2.7	7.2	109.6	100.2	4 h at RT
			(0.029/0.089) *						
UV-BCA	MCH	0.031–2.000	0.008/0.024	0.9966	1.6	5.4	106.7	106.3	<10 min
	BCH		0.009/0.027	0.9988	2.7	5.2	107.9	107.5	
FLD-OPA	MCH	0.005–0.075	0.0009/0.0028	0.9958	3.3	32.4	106.6	104.3	<10 min
	BCH		0.0010/0.0029	0.9987	3.9	32.6	103.8	100.9	

* Approximate corresponding collagen concentrations. Abbreviations: MCH—marine collagen hydrolysate standard; BCH—bovine collagen hydrolysate standard; GPH—tripeptide Gly-Pro-Hyp; Hyp—hydroxyproline; LOD—limit of detection; LOQ—limit of quantification; R^2^—determination coefficient; RT—room temperature.

**Table 2:** Content of collagen in commercially available food supplements.

Product	Content ± SD [%]							
	HPLC-CH	HPLC-GPH	HPLC-dNGP	FLD-dNGP	HPLC-dHyp	UV-dHyp	UV-BCA	FLD-OPA
A	97.8 ± 2.5	100.1 ± 1.5	95.0 ± 2.5	91.9 ± 4.8	107.1 ± 5.7	99.1 ± 15.4	185.7 ± 5.4	70.8 ± 5.8
B	100.6 ± 5.0	109.0 ± 2.1	95.7 ± 4.7	94.1 ± 3.0	95.9 ± 0.6	111.9 ± 2.8	225.8 ± 13.6	94.8 ± 0.6
C	98.4 ± 2.4	105.4 ± 1.3	100.7 ± 1.5	90.8 ± 2.8	92.4 ± 0.4	93.1 ± 12.6	171.2 ± 4.2	70.3 ± 2.8
D	103.8 ± 2.8	107.8 ± 5.3	104.7 ± 3.5	94.8 ± 9.7	113.4 ± 14.3	95.7 ± 8.4	140.3 ± 6.5	103.1 ± 8.7
E	106.5 ± 0.9	114.0 ± 2.0	99.2 ± 5.8	93.5 ± 8.4	105.1 ± 8.7	96.0 ± 9.7	234.7 ± 17.7	104.7 ± 2.2
F	98.5 ± 0.4	108.0 ± 6.4	104.5 ± 2.4	91.8 ± 2.7	117.9 ± 2.7	95.7 ± 8.4	135.2 ± 1.5	96.5 ± 3.7
G	95.8 ± 4.6	100.4 ± 1.6	99.7 ± 5.5	95.2 ± 4.2	101.8 ± 10.9	99.9 ± 2.2	256.7 ± 16.0	94.6 ± 5.5
H	101.6 ± 0.5	112.9 ± 1.0	84.3 ± 1.9	77.4 ± 5.1	87.5 ± 4.2	94.6 ± 14.9	180.5 ± 8.3	104.9 ± 5.7
I	99.6 ± 1.7	88.8 ± 2.2	86.7 ± 4.0	81.7 ± 6.3	95.7 ± 3.3	97.8 ± 13.7	175.8 ± 6.6	102.9 ± 3.6
J	106.6 ± 0.7	112.3 ± 0.9	93.2 ± 3.5	92.9 ± 2.7	101.0 ± 5.3	109.3 ± 5.8	107.1 ± 1.6	106.5 ± 1.7
Range of content	95.8–106.6	88.8–114.0	84.3–104.7	77.4–95.2	87.5–117.9	93.1–111.9	107.1–256.7	70.3–106.5
Average recovery	103.2%	90.8%	91.5%	95.5%	96.6%	99.1%	89.5%	89.0%
Average RSD	2.2%	2.3%	3.8%	5.5%	5.4%	9.7%	4.1%	4.4%

**Table 3:** Origin of collagen in commercially available food supplements.

Product	Labeled Origin	Content of Gly-Pro-Hyp ± SD [%]	Identified Origin *
A	marine	3.62 ± 0.06	marine
B	bovine	4.61 ± 0.09	terrestrial
C	marine	3.81 ± 0.05	marine
D	porcine	4.42 ± 0.22	terrestrial
E	marine, bovine, chicken	4.62 ± 0.08	terrestrial
F	marine	3.90 ± 0.25	marine
G	marine	3.71 ± 0.06	marine
H	no information	4.73 ± 0.04	terrestrial
I	marine	3.11 ± 0.08	marine
J	bovine	4.48 ± 0.03	terrestrial
Standard	marine	3.84 ± 0.02	
	bovine	4.61 ± 0.06	

* Terrestrial: bovine, porcine, chicken, etc.

**Table 4:** Comparison of tested methods for evaluation of collagen content in food supplements.

	Specificity	Linearity Accuracy Precision	Sample Stability	Complexity of Sample Preparation	Quality Control of Food Supplements
HPLC-CH	+	+	++	++	+
HPLC-GPH	++	+	++	+	++
HPLC-dNGP	+/−	+	−	+/−	+
FLD-dNGP	+/−	+/−	+/−	+/−	+
HPLC-dHyp	+	+	+	−	+
UV-dHyp	+	+	+	−	+/−
UV-BCA	−	+	−	+	−
FLD-OPA	−	−	−	+	−

Legend: (++): best method; (+): method meets specified criteria; (+/−): method has advantages and disadvantages; (−): method has drawbacks

**Table 5:** Additional data on tested food supplements containing collagen.

	Product Name and Manufacturer	Dosage Form	Type of Collagen	Labeled Content of Collagen	Other Ingredients
A	Collagen Skin Care (Nutrisslim d.o.o., Ig, Slovenia)	Powder	Hydrolyzed marine collagen (NatiCol^®^)	50.0 g/100 g	Ashwagandha, MSM, vitamin C
B	Collagen Joint Care (Nutrisslim d.o.o., Ig, Slovenia)	Powder	Hydrolyzed bovine collagen (Fortigel^®^)	50.0 g/100 g	MSM, rosehip
C	Kolagen lift (Medex d.o.o., Ljubljana, Slovenia)	Powder	Hydrolyzed marine collagen (NatiCol^®^)	62.5 g/100 g	Hyaluronic acid, inulin, beetroot, vitamin C
D	Skin beauty biotin collagen (Vitabalans d.o.o., Ljubljana, Slovenia)	Powder	Hydrolyzed porcine collagen type I (Verisol^®^ P)	92.6 g/100 g	Vitamins B7 and C, hyaluronic acid
E	Collagen powder blend (Biovis d.o.o., Komen, Slovenia)	Powder	Hydrolyzed collagens from different sources: bovine type I and II, marine type I, chicken type II	84.3 g/100 g	Shilajit, curcumin, eggshell membrane, silica, vitamin C
F	Kolagen (Valens d.o.o., Komenda, Slovenia)	Oral solution	Hydrolyzed marine collagen (Peptan^®^)	26.7 g/100 mL	Vitamins A, B7, C, zinc, coenzyme Q10, selenium
G	Skinage advanced (Yasenka d.o.o., Vukovar, Croatia)	Oral solution	Hydrolyzed marine collagen	20.0 g/100 mL	Vitamins B3 and C, zinc, hyaluronic acid
H	Kolagen 820 + Hialuron + Q10 (Queisser Pharma GmbH & Co., Flensburg, Germany)	Capsule	Hydrolyzed collagen	0.82 g/capsule	Copper, coenzyme Q10, manganese, vitamins B7 in C
I	Kolagen (Multinorm, Ljubljana, Slovenia)	Oral solution	Hydrolyzed marine collagen	26.7 g/100 mL	Coenzyme Q10, vitamins A, C and B7, zinc, selenium
J	Kolagen flex (Medex d.o.o., Ljubljana Slovenia)	Powder	Hydrolyzed bovine collagen (Fortigel^®^)	100.0 g/100 g	Vitamin C

## Data Availability

Data are contained within the article; further inquiries can be directed to the corresponding author.
